# Towards global insect biomonitoring with frugal methods

**DOI:** 10.1098/rstb.2023.0103

**Published:** 2024-06-24

**Authors:** Mikkel Brydegaard, Ronniel D. Pedales, Vivian Feng, Assoumou saint-doria Yamoa, Benoit Kouakou, Hampus Månefjord, Lorenz Wührl, Christian Pylatiuk, Dalton de Souza Amorim, Rudolf Meier

**Affiliations:** ^1^ Dept. Physics, Lund University, Sölvegatan 14c, 22362 Lund, Sweden; ^2^ Dept. Biology, Lund University, Sölvegatan 35, 22362 Lund, Sweden; ^3^ Norsk Elektro Optikk, Østensjøveien 34, 0667 Oslo, Norge; ^4^ FaunaPhotonics, Støberi Støberigade 14, 2450 København, Denmark; ^5^ Institute of Biology, University of the Philippines Diliman, Quezon City, Philippines 1101; ^6^ Center for Integrative Biodiversity Discovery, Museum für Naturkunde, Leibniz Institute for Evolution and Biodiversity Science, Invalidenstraße 43, 10115, Berlin, Germany; ^7^ Institute of Biology, Humboldt University, 10115 Berlin, Germany; ^8^ Instrumentation, Imaging and Spectroscopy Laboratory, Felix Houphouet-Boigny Institute, BP1093 Yamoussoukro, Ivory Coast; ^9^ Institute for Automation and Applied Informatics, Karlsruhe Institute of Technology, 76344 Eggenstein-Leopoldshafen, Germany; ^10^ Departamento de Biologia, FFCLRP, Universidade de São Paulo, Ribeirão Preto 14040-901, Brazil

**Keywords:** insect biomonitoring, frugal science, photonics

## Abstract

None of the global targets for protecting nature are currently met, although humanity is critically dependent on biodiversity. A significant issue is the lack of data for most biodiverse regions of the planet where the use of frugal methods for biomonitoring would be particularly important because the available funding for monitoring is insufficient, especially in low-income countries. We here discuss how three approaches to insect biomonitoring (computer vision, lidar, DNA sequences) could be made more frugal and urge that all biomonitoring techniques should be evaluated for global suitability before becoming the default in high-income countries. This requires that techniques popular in high-income countries should undergo a phase of ‘innovation through simplification’ before they are implemented more broadly. We predict that techniques that acquire raw data at low cost and are suitable for analysis with AI (e.g. images, lidar-signals) will be particularly suitable for global biomonitoring, while techniques that rely heavily on patented technologies may be less promising (e.g. DNA sequences). We conclude the opinion piece by pointing out that the widespread use of AI for data analysis will require a global strategy for providing the necessary computational resources and training.

This article is part of the theme issue ‘Towards a toolkit for global insect biodiversity monitoring’.

## 1. Introduction

Of the 20 global biodiversity conservation targets for 2020 (Aichi Targets, United Nations), only six have been partially achieved (see [1]). At the same time, one-third of the UN Decade on Ecosystem Restoration (spanning from 2021 to 2030) has passed, and humanity is not even collecting enough data to identify areas in need of restoration. The available data indicate fast decline in biodiversity [2,3], but the information is biased because many biomonitoring programmes still prioritize the charismatic megafauna [4], while the biomass and diversity of arthropods are 100 times greater than that of all wild birds, mammals, amphibians and reptiles combined [5]. Holistic biomonitoring undoubtedly has to include insects [6,7] but should not stop at bees and butterflies.

The taxon-biases in the available data are bad enough, but there is a second problem that is at least as serious. It is the failure to gather enough biodiversity data for those countries that are home to the largest number of species. In particular, the lack of data for most of the tropics is deeply worrying, because 14 of the 17 megadiverse countries are located in what is sometimes called the ‘Global South’ [8,9]. Many of these countries lack biodiversity baseline data, let alone biomonitoring capacity [10,11]. The full extent of the problem is unknown, but the Catalogue of Life (accessed 4 September 2023) lists 970 814 described species of insects in the world [12], although Stork [13] estimates that the number of insect species in the Afrotropical, Indo-Malayan, Neotropical and Oceanic regions alone exceeds 4 million. However, of the 163 million occurrence data points for insects in GBIF (Global Biodiversity Information Facility: https://www.gbif.org/, accessed 11 September 2023) only 18.9% (31 million) pertain to these 17 megadiverse countries. Moreover, a mere 3.45% of GBIF's insect data (5.6 million) lie within the borders of the aforementioned 14 megadiverse countries. The root cause for this lack is a mixture of financial constraints, funding priorities and the high cost of data acquisition and analysis. The result is a disparity between capacity and data requirements for many biodiverse countries [14,15]. Of course, this problem is exacerbated when expensive biomonitoring methods and technologies are adopted because they divert funds away from more frugal options. In this opinion paper, we use examples from different fields to argue that insect biomonitoring will have to adopt frugal methods to achieve global reach.

## 2. Frugal science

All scientific methods need to yield accurate results, but only recently more attention has been paid to developing tools that also emphasize affordability, accessibility and sustainability (referred to as ‘frugal science’). Frugal science is urgently needed for the study of biodiversity because the knowledge gaps regarding ‘dark taxa’ alone are so vast that ‘non-frugal’ science is not an option. Indeed, more than 50% of all insect species in mass samples belong to taxa that are taxonomically neglected worldwide [16,17]. An example is phorid flies, for which a single Malaise trap placed in an African secondary forest can yield more than 650 species although only 466 species have been described for the entire Afrotropical region [17]. This means that we currently have dangerous knowledge gaps that will be difficult to fill without frugal methods. Unfortunately, these knowledge gaps tend to grow the closer one gets to the equator, but they are also a serious problem for all temperate and subtropical regions.

The development of frugal methods generally starts with an initial phase when existing instruments and methods are simplified using the mind-set of ‘low cost but good enough’ [18]. Frugal science thus tries to overcome the tendency of many researchers to confuse 'complicated' with 'sophisticated'. Indeed, there is evidence that innovation through subtraction is systematically overlooked in science [19]. This oversight artificially inflates the cost of science. In order for simplification to be successful, it is important that all methods and construction plans for equipment are open access. This is because collaborative development and modifications are facilitated when blueprints and part lists of a technology are comprehensively described. Another important aspect is the suitability of a technique for utilizing locally sourced equipment and consumables instead of imported goods. This is important because it empowers researchers to be independent and eliminates import-related costs and delays. Historically, such local sourcing often meant co-opting mass-produced parts that were initially produced for a different purpose. Examples are DNA purification and preservation with paper towels at room temperature [20,21], the use of an orbital shaker for size-sorting insect samples constructed from mostly household goods [22], or the use of tea strainers for the safe transport of Malaise trap samples [23], but nowadays co-option can be complemented with three-dimensional printing of purpose-built designs because three-dimensional printers are now ubiquitous.

Frugal approaches to insect biomonitoring are still underdeveloped, but several recent developments are opening the flood gates. As part of the open science movement, methods and equipment are described in more detail, which helps with applying innovation through subtraction. A second development is the boom in consumer electronics and affordable three-dimensional printing. Just consider photonics, where the consumer devices range from inexpensive laser pointers, laser printers and projectors to fibre routers and BlueRay DVD drives. Many of these devices include high-power lasers covering wavelengths from violet to infrared. Photodetectors can be found in routers, flatbed scanners and cameras integrated into smartphones and tablets. Such products can be scavenged for parts, hacked or used in their entirety, also making use of their auxiliary features such as GPS location and wireless data transmission. Creative use and hacking of advanced technology are getting increasingly feasible since enthusiasts can compare experiences, code and technical drawings in online forums such as *YouTube*, *GitHub* and *Global Open Source Hardware* initiatives (GOSH). In addition, designs can now be shared, modified and/or replicated [24] through three-dimensional printing and automated measurement systems can be designed using robotic kits such as *LEGO Mindstorms*, *Raspberry Pi* or *Arduino*. Whereas research groups in countries with high salary costs hesitate to spend on reverse engineering, researchers in other countries and enthusiasts in entomological societies are often ready to spend time instead of money.

## 3. Emerging technologies in insect biomonitoring: 1. Computer vision

**Figure 1.**
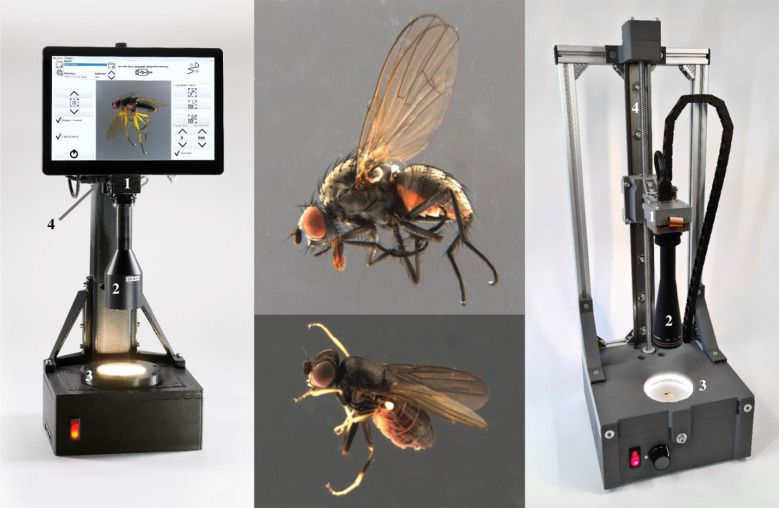
Standalone entomoscope (left) and plug-in entomoscope (right) featuring a 12 MP low-cost camera (1) adaptable to various C-/CS-mount lenses (2) to accommodate specimens of different sizes. Specimens are positioned within a Petri dish, illuminated by a ring light at the periphery (3). Both entomoscopes are equipped with a linear stage (4) for precise focusing and focus stacking, allowing vertical camera movement [30]. Two images taken with an entomoscope are shown in the middle.

Arguably, the frugal use of computer vision is the most promising of the technologies for insect biomonitoring [25]. Two reasons are the low cost and the ubiquitous availability of digital colour cameras in smartphones. This has already led to the development of many mobile phone apps for species recognition based on machine learning, such as *Seek*, *Picture Insect* and *Google Lens*. The potential is vast because image recognition can be combined with automated surveillance of flower patches [26] or light traps [27]. However, the algorithms are overwhelmingly trained for charismatic taxa occurring in the Northern temperate regions [28,29]; i.e. there tends to be a negative relationship between the species diversity of a region/taxon and the availability of trained algorithms. Overcoming these biases will be paramount to success, but likely require different strategies for charismatic and non-charismatic taxa. The latter have particularly high species numbers [16], but resolving species diversity and abundance will likely need a combination of images and inexpensive DNA barcodes obtained with new sequencing technologies for assigning the images to species-level units. It is here that the availability open-access camera systems is particularly important because they allow for applying DIY (Do it Yourself) principles. The newly developed ‘entomoscope’, for example [30], is a low-cost, open-source photomicroscope for taking high-resolution, focus-stacked images that are suitable for training AI algorithms (figure 1). The software and construction plans are open access, with the main body of the microscope being three-dimensional-printed. The remaining parts can be bought off-the-shelf or substituted by locally available parts. Entomoscopes were developed because both DIY and commercially available microscopes struggled with imaging specimens in ethanol, which is the most popular preservative for insect mass samples because it is widely available, inexpensive and preserves DNA. These samples are dominated by small hyperdiverse insect taxa [16] that needed an imaging solution. Imaging these insects can be combined with robotic specimen handling and focus stacking, which greatly increase the quality of the photographs [31,32]. Similarly, motorized cameras can be used to generate images from a sufficient number of angles to generate three-dimensional models for the purpose of quantitative morphological study [31,33].

In comparison to imaging small specimens preserved in ethanol, the photography of larger and even living insects is well established and images are shared in large numbers on websites such as iNaturalist (https://www.inaturalist.org/). However, more data for charismatic insects could be obtained by further simplifying data acquisition and the introduction of game playing elements (gamification), allowing very active users to earn reward badges (see the *Seek* app developed by iNaturalist: https://www.inaturalist.org/pages/seek_app). To increase the number of observers, cost-effective open-source DIY digital microscopes can be built from two identical smartphones: the optics are salvaged from one and then placed in reverse in front of the other [34]. This ensures perfect 1:1 imaging from sample to the imaging chip at high resolution given that the pixel sizes in smartphones are typically below 2 µm. The instruments needed for three-dimensional modelling are not expensive, because this technique can yield inexpensive but detailed three-dimensional models of insects in ‘natural colours’ given that motorized automation can now use three-dimensional printing, *Raspberry Pi* and *LEGO* [35]. This could be a starting point for a real-world version of *Pokémon Go* (pocket monsters) originally invented by a Japanese media company. However, the gamification of insect biomonitoring requires careful planning because currently not even many charismatic tropical species can be identified with AI algorithms. This means that gamification must also cover species discovery, where photographing insects is followed by collecting vouchers for identification.

**Figure 2.**
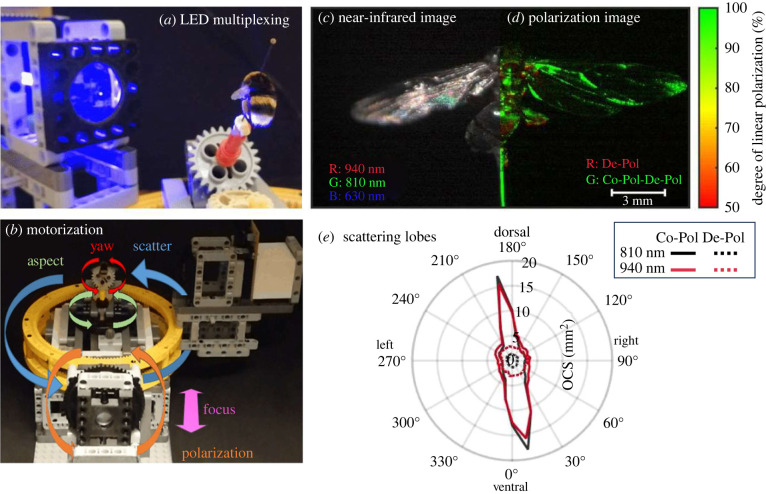
(*a*) Low-cost multispectral imaging of insects by LED multiplexing. (*b*) Adding dimensionality to insect scanning with a robotic LEGO® kit. (*c*) False colour near-infrared images allow quantification of the melanin and chitin pathlengths with nanometer precision, (*d*) Polarimetric imaging allow assessment of how many times the photons scatter in the sample, (*e*) Angular stages allow projection of insects from all sides or investigation of scattering lobes relating to nanofeatures such as surface roughness.

**Figure 3.**
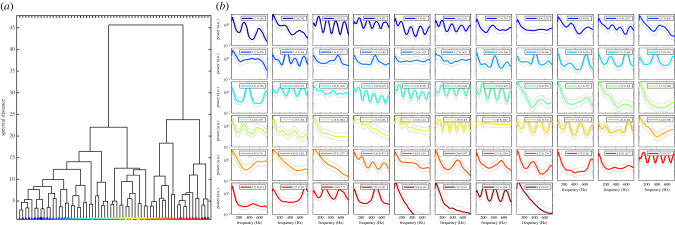
The diversity of hundreds of thousands oscillatory signals collected by lidar is expected to reflect the species richness. (*a*) Hierarchical clustering of 58 499 insect signals collected in a 145 m lidar transect over a rice field in Yamoussoukro, Ivory Coast. (*b*) Corresponding power spectra with within-group variance (grey lines) of each branch in the dendrogram in part (*a*). 'C' denotes the cluster number and 'N' denotes number of observations within the cluster.

But why stop at only using the human spectral bands for biomonitoring insects? Insects perceive a very different world because they use up to six bands from UV to deep red [36], with some species also distinguishing polarizations [37]. With minimal effort, LEDs or laser diodes can be multiplexed [35] to capture insects using their own vision bands (figure 2*a*). And while we are at it, why not automate polarization and scatter angle [38,39]? Adding degrees of freedom to insect photography allows for the acquisition of millions of unique pictures (figure 2*b*). Entering the world of biophotonics allows for exploring insects' manipulation of light by nanofeatures (e.g. extreme black- and whiteness [40,41]), chirped mirrors in beetles displaying goldish appearance [42], iridescence and directional reflectance in butterflies [43] or circular dichroism by chiral nanostructures in beetles [44]. Surely recording the light-scattering from unstudied insect species in the tropics could lead not only to the discovery of new species but also to more appreciation and novel insights into how light can be manipulated. For example, light-scattering lobes from diffuse wings of moths can be associated with sub-resolution surface roughness. Inexpensive near-infrared photographs (figure 2*c*) allow for quantification of the equivalent absorption path length of melanin in insect cuticles. Similarly, spectral imaging of wing interference patterns (WIPs) can be done with inexpensive instruments [35] and the thickness of the chitin membrane can be quantified in each pixel [45] with a confidence interval in the order of 10 nm. It is conceivable that wing morphology [46] and quantitative WIPs patterns [47] alone would be sufficient for identifying particularly closely related insect species at a lower cost than alternative techniques such as DNA barcoding (figure 3).

## 4. Emerging technologies in insect biomonitoring: 2. Lidars and photonic sensors

Ideally, insect populations should be assessed continuously, at low cost, and without harming the populations. Unfortunately, motion blur makes photographing insects in flight challenging. Detecting and classifying insects during flight requires a different focus strategy such as the Scheimpflug principle [48,49] or post-focusing by digital holography [50]. Probably the best solution is relying on domains that do not defocus, such as oscillation frequencies, spectral wavelengths or light polarization. This can be surprisingly frugal, because wing beat frequencies can even be captured by the microphone of a smartphone. Indeed, this has been proposed for monitoring mosquitoes in the bedroom while the phone is charging [51]. Other insect taxa produce signals that can be acquired by connecting a photodiode with a transimpedance amplifier to the audio input of a recording device [52]. Soldering together these three components only costs a couple of dollars. For minimal costs, carrier frequencies or multiplexing can be implemented to remove background and measure backscatter in multiple wavelengths, for example to quantify melanization. For example, several companies have proposed insect monitoring and classification based on such wing beat sensors [53–56]. Considering a relative within-species spread of wingbeat frequency of approximately 25%, one could distinguish as many wingbeats as there are notes on a two-octave piano. Of course, thousands of species can coexist in a habitat, but they can conceivably be distinguished by overtones. Such optical overtones relate the nanometre wing interference (see above).

A particular frugal approach to optical monitoring of living insects in flight is to expand and collimate a laser diode to create a probe volume of hundreds of metres. With comparable low laser power of a couple of watts, light can be recycled metre after metre until it intercepts an insect. The backscattering from such an elongated probe volume can be sharply imaged onto a fast linear imaging chip by the Scheimpflug principle. In this way, hundreds of thousands of insects can be detected per day and classified according to their oscillatory properties. By making use of commercially available large optics from amateur astronomy, infrared laser diodes, linear digital cameras and 3D printed instrumentation [57], any enthusiastic hobbyist could build their own entomological lidar in a garage. Research-grade entomological lidar of varying complexity and numbers of bands have been deployed in biodiverse countries such as China [58], Tanzania [59], Ivory Coast [60], Ecuador [49], Colombia and the USA (Texas) [61]. The one-time cost of these systems is in the order of $10 000, but they can generate data continuously and at a very high rate. For example, 58 499 insect signals were detected over an Ivorian rice field in a single day (figure 3). The unique signals are found by hierarchical clustering [60] and some clusters, like C52 and C59 in figure 3*b*, can be associated with female and male mosquitoes, respectively. The cost per observation is 10 cents the first day and free for the following days. Lidar and photonics sensors are thus interesting frugal techniques for insect monitoring. All required information is open access and the equipment cost is manageable. However, widespread adoption will require user-friendly data analysis pipelines and more systematic matching between oscillatory lidar signals and species.

## 5. Emerging technologies in insect biomonitoring: 3. Genetic methods

Over recent decades, DNA barcoding and metabarcoding have revolutionized biomonitoring by adding molecular identification tools to the existing repertoire based on morphology [62]. Much information on the biodiversity of rich countries located in temperate regions has been collected [63–66], but mid- and low-income countries have been struggling [67]. For example, the Philippines is in the process of building a comprehensive DNA barcode database for its rich biodiversity to address pronounced taxonomic and spatial data biases [68,69]. However, the high cost of sequencing poses significant barriers [70] given that the cost of DNA barcoding including labour has been estimated to be $5 per sample in the USA [71], while it is $48 in the Philippines. Furthermore, all imported instruments and consumables are expensive owing to customs regulations, high shipping costs and reliance on local distributors who charge a premium [72].

Arguably, there is bad and good news with regard to frugal DNA-based biomonitoring methods. The bad news is that commercial sequencing technologies have high consumable costs, consumables have to be imported into most low- and mid-income countries and most sequencers have very high capital costs. None of this is likely to change unless one of the producers of sequencers were to decide to price according to income levels. The good news is that there are two obvious ways to avoid high sequencing cost in countries with high import tariffs. The first is sending samples abroad, but this generates undesirable dependencies and much paperwork. The second way is using techniques that do not require high read coverage for samples. We believe that this favours barcoding of individual specimens over metabarcoding of the DNA extracted from entire samples with thousands of specimens, given that the latter requires higher sequence coverages to accommodate body size differences. Fortunately, high-throughput barcoding of individual specimens (‘megabarcoding’ [73]) has become much more affordable because there are now frugal techniques that, for example, avoid DNA extraction, allow for multiplexing a very large number of samples on one flowcell and embrace affordable sequencers such as the MinION [74]. Furthermore, megabarcoding can become unnecessary over time because it allows for reaching a low-cost, sustainable state of insect biomonitoring; i.e. species identification based on images. For reaching this stage, specimens are first imaged (e.g. with a DIY microscope) and then barcoded. Image training sets for AI algorithms are then obtained by grouping the images according to putative species defined based on barcodes. Once trained, specimens belonging to many common species can be identified based on images and no longer have to be sequenced.

Many of the other costs associated with preparing samples for sequencing can also be dramatically reduced. There are DIY plans for building most of the required instruments. This includes thermocyclers and centrifuges [75], but also consumables such as racks [76] and enzymes [77]. Moreover, plastic consumables and agarose can be reused during megabarcoding because it is much less sensitive to contamination than metabarcoding. All of this is not only frugal but it also reduces the amount of waste generated by molecular labs [78]. Indeed, a rough estimate is that in 2014 approximately 5.5 million tonnes of plastic waste were generated by biological, medical or agricultural research laboratories [79]. Much of it was single-use plastics, although some consumables could have been reused [80,81]. Overall, it appears to us that molecular biomonitoring is currently far from being in line with the UN′s Sustainable Development Goals (SGDs; e.g. environmental, social and economic SDGs) and the development of frugal approaches is a must. To achieve this, we urgently need answers to a range of questions: How can the dependency on patented sequencing technologies be reduced? Why is there still so much use of plastic consumables and how can we promote the recycling of consumables? How can metabarcoding be made frugal given that it is such an important technique for so many purposes?

## 6. Conclusion

The widening gap in research capacity between countries with very different science funding [82] can only be overcome if more biomonitoring techniques are frugal. Research and technologies must be made accessible to more citizens and to empower communities and countries to manage their biodiversity. Particularly attractive will be identification apps using images taken by mobile phones. Particularly unattractive will be one-way practices such as shipping samples halfway across the globe and then paying for sample processing and voucher return (https://ccdb.ca/pricing/). Fortunately, frugal science and open access have the potential to alleviate dependency on well-equipped facilities abroad and pave the way for better international collaborations. However, one challenge will be hard to overcome even if all data acquisition methods have been made frugal. This is data analysis and AI model training, which require expensive software and significant computational resources. There are few free options (e.g. *Google Colaboratory*) but they have tight usage limits. Popular paid platforms such as *Amazon Web Services* are expensive and require reliable high-throughput internet connections that are often unavailable in areas with particularly high biodiversity. High-income countries should start supporting initiatives that generate access to data management and analysis infrastructure. At least as important will be the creation of training programmes in data analysis.

## Data Availability

This article has no additional data.
